# Undescended Testis in a Zambian Cadaver: A Cadaveric Case Report With Anatomical and Clinical Correlation

**DOI:** 10.7759/cureus.103508

**Published:** 2026-02-12

**Authors:** Sunita Sethy, Amit Kumar Singh, Vivienne Nambule Syamuleya, Ryan Nkhoma, Wellington Simango, Krishna M Jasani, Krupal J Joshi

**Affiliations:** 1 Anatomy, Texila American University Zambia, Lusaka, ZMB; 2 Community and Family Medicine, All India Institute of Medical Sciences, Rajkot, IND

**Keywords:** clinical anatomy, cryptorchidism, inguinal canal, the cadaveric study, undescended testis

## Abstract

Undescended testis (UDT) (cryptorchidism) is the most common congenital anomaly of male genital development. While typically identified and managed in infancy, persistence into adulthood is rare and infrequently documented through anatomical studies, particularly in African populations. During routine undergraduate anatomical dissection at Texila American University, Lusaka, an embalmed adult male cadaver was examined. Detailed dissection of the inguinal canal and scrotal contents revealed a true right-sided undescended testis located within the inguinal canal. The testis was morphologically intact and supplied by normally arranged testicular vessels, vas deferens, and pampiniform plexus. No evidence of testicular atrophy, fibrosis, malignancy, or prior surgical intervention was identified. The contralateral testis was normally positioned within the scrotum. This cadaveric case report documents a rare adult presentation of persistent unilateral inguinal cryptorchidism in a Zambian male. The finding highlights the anatomical and clinical significance of undiagnosed cryptorchidism and underscores the importance of early detection and timely surgical intervention to prevent long-term complications.

## Introduction

Cryptorchidism, or undescended testis (UDT), is the most prevalent congenital anomaly affecting male genital development. It is defined as the failure of one or both testes to descend into the scrotum by birth or during the early postnatal period. The reported prevalence ranges from 3% to 5% in full-term male neonates and up to 30% in preterm infants, with spontaneous descent occurring in approximately 70%-75% of cases within the first year of life in the African population [1]. Persistence beyond infancy is clinically significant due to its association with infertility, torsion, inguinal hernia, and testicular malignancy [1].

Normal testicular descent occurs in two hormonally mediated phases. The transabdominal phase is primarily regulated by insulin-like peptide 3 (INSL3), while the inguinoscrotal phase depends on testosterone, Müllerian inhibiting substance (MIS), and genitofemoral nerve-derived calcitonin gene-related peptide signaling [2]. Arrest at any stage may result in cryptorchidism, with the inguinal canal being the most frequent site of testicular retention [3].

Although cryptorchidism is extensively documented in pediatric and clinical populations, reports of persistent undescended testes in adults are uncommon. Anatomical documentation through cadaveric studies is particularly rare, especially in sub-Saharan Africa where epidemiological data are limited. Cadaveric case reports provide a unique opportunity to examine long-standing anatomical adaptations and contribute region-specific evidence to the existing literature. This report describes an incidental finding of persistent unilateral inguinal cryptorchidism in an adult Zambian cadaver and discusses its anatomical and clinical relevance.

## Case presentation

This cadaveric case report was conducted in the dissection laboratory of Texila American University, Lusaka. During routine undergraduate anatomical dissection, an embalmed adult male cadaver was examined. Ethical approval for the use of cadaveric material in teaching and research was obtained prior to the study (AUZ/REC/2025/F/09).

Dissection was performed using standard anatomical techniques in accordance with Grant’s Dissector [4]. The inguinal canal, spermatic cord, and scrotal contents were exposed bilaterally in a cadaver. Observations focused on testicular position, morphology, vascular supply, and continuity of the vas deferens. Relevant findings were documented photographically.

The inguinal canal, spermatic cord, and scrotal contents were carefully exposed. There was no external or internal evidence of prior inguinal surgery, orchidopexy, trauma, or post-embalming displacement.

**Figure 1:**
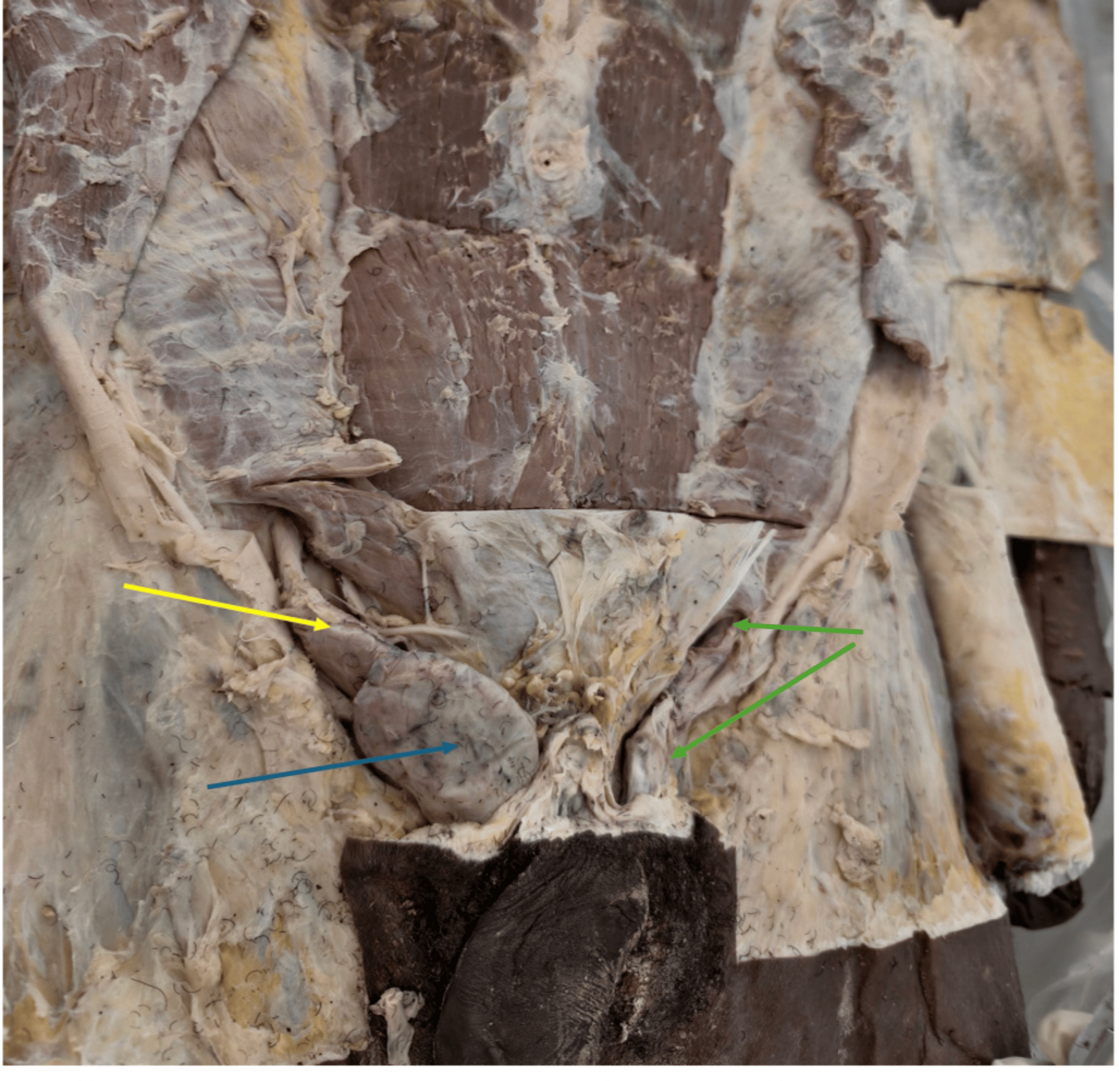
Right undescended testis located in the inguinal canal Dissection of the inguinal region showing the right spermatic cord (yellow arrow), the undescended right testis (blue arrow), and the left inguinal canal (green arrow).

**Figure 2:**
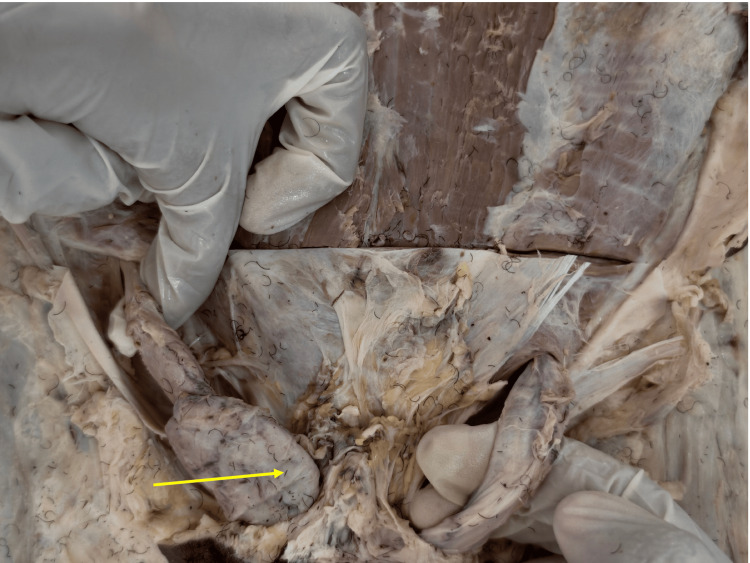
Spermatic cord structures associated with the undescended testis Close-up view demonstrating the spermatic cord structures of the undescended right testis, including the testicular artery, pampiniform plexus, and vas deferens (arrow), all showing normal anatomical continuity without evidence of prior surgical intervention.

On examination, the right testis was absent from the scrotum and was located within the inguinal canal, consistent with true inguinal cryptorchidism (Figure 1). The testis was ovoid, well-formed, and comparable in size to a normally descended adult testis. The spermatic cord structures, including the testicular artery, pampiniform plexus, and vas deferens, were intact and demonstrated normal anatomical continuity (Figure 2). No macroscopic evidence of testicular atrophy, fibrosis, or malignancy was observed. The surrounding inguinal canal structures were intact, supporting a congenital undescended state rather than secondary displacement. The left testis was normally positioned within the scrotum and exhibited typical adult morphology.

Based on anatomical location and appearance, the finding was classified as a true unilateral inguinal undescended testis, rather than an ectopic, retractile, ascending, or vanishing testis [3].

## Discussion

Persistent cryptorchidism into adulthood is rare, as most cases either descend spontaneously during infancy or are surgically corrected in early childhood. The present cadaveric case demonstrates a long-standing undescended testis that remained untreated, likely reflecting delayed diagnosis or limited access to pediatric surgical care. Kolon et al. [1] report that nearly 80% of cases are identified at birth, with spontaneous descent in most infants within the first year. Failure of descent beyond this period increases the risk of long-term complications.

The anatomical findings in this case align with established mechanisms of testicular descent failure. Disruption of INSL3-mediated gubernacular development or androgen-dependent inguinoscrotal migration can result in arrest within the inguinal canal [2,5], which remains the most frequently reported site of undescended testes [3]. The preserved morphology and vascular supply observed in this case suggest that significant degenerative changes are not inevitable, although functional impairment cannot be excluded.

Persistent cryptorchidism exposes the testis to suprascrotal temperatures, leading to progressive degeneration of germinal epithelium, impaired spermatogenesis, and an increased risk of testicular malignancy, particularly seminoma [6,7]. Although histopathological examination was not performed, the presence of an undescended testis in adulthood represents a recognized high-risk clinical condition.

Reports of adult cryptorchidism from African populations are scarce, likely due to underdiagnosis, underreporting, and limited access to early surgical intervention [8]. Therefore, the presence of this anomaly in an adult cadaver suggests a lack of early diagnosis or limited access to pediatric surgical care. Comparable gaps in the detection of congenital urogenital anomalies have been reported in African populations [9], supporting the likelihood of regional under-reporting.

This cadaveric case contributes region-specific anatomical evidence and highlights the role of anatomical dissection in identifying overlooked congenital anomalies. Current clinical guidelines consistently recommend early orchidopexy, ideally within the first year of life, to preserve fertility potential and reduce malignancy risk [10,11]. The present finding underscores the anatomical consequences of delayed or absent intervention.

This report is limited by its nature as a single cadaveric observation, which restricts generalizability and precludes estimation of prevalence or risk factors for persistent cryptorchidism in the broader population. As the demographic, clinical, and surgical history of the individual was unavailable, correlations with fertility status, hormonal profile, or lifetime clinical outcomes could not be established. Additionally, histopathological examination of the undescended testis was not performed, limiting assessment of microscopic changes such as germ cell loss, dysplasia, or early malignant transformation. Despite these limitations, the case provides valuable anatomical insight into a rare adult presentation of cryptorchidism and contributes region-specific evidence from an under-reported population.

## Conclusions

This cadaveric case report documents a rare anatomical finding of persistent unilateral inguinal cryptorchidism in an adult Zambian male. Although limited to a single case, the observation contributes valuable regional data to an under-documented condition. The findings reinforce the importance of early diagnosis, timely surgical correction, and improved surveillance of congenital urogenital anomalies, particularly in resource-limited settings.
